# Herpes Simplex Virus (HSV) Modulation of *Staphylococcus aureus* and *Candida albicans* Initiation of HeLa 299 Cell-Associated Biofilm

**DOI:** 10.1007/s00284-015-0975-7

**Published:** 2016-01-13

**Authors:** Balbina J. Plotkin, Ira M. Sigar, Vaibhav Tiwari, Scott Halkyard

**Affiliations:** Department of Microbiology and Immunology, Midwestern University, Downers Grove, IL 60515 USA

## Abstract

Although herpes simplex virus type-1 (HSV-1), and type-2 (HSV-2), *Staphylococcus aureus* and *Candida albicans* co-habit the oral and genital mucosa, their interaction is poorly understood. We determined the effect HSV has on bacterial and/or fungal adherence, the initial step in biofilm formation. HeLa229 cells were infected with HSV-1 (KOS) gL86 or HSV-2 (KOS) 333gJ^**−**^ at a multiplicity of infection (MOI) of 50 and 10. *S. aureus* (ATCC 25923) and/or *C. albicans* (yeast forms or germ tube forms) were co-incubated for 30 min (37 °C; 5 % CO_2_; 5:1 organism: HeLa cell ratio; *n* = 16) with virus-infected HeLa cells or uninfected HeLa cell controls. Post-incubation, the monolayers were washed (3x; PBS), lysed (RIPA), and the lysate plated onto Fungisel and/or mannitol salts agar for standard colony count. The level of HeLa-associated *S. aureus* was significantly decreased (*P* < 0.05) for both HSV-1- and HSV-2-infected cells, as compared to virus-free HeLa cell controls (38 and 59 % of control, respectively). In contrast, HSV-1 and HSV-2 significantly (*P* < 0.05) enhanced HeLa cell association of *C. albicans* yeast forms and germ tube approximately two-fold, respectively. The effect of *S. aureus* on germ tube and yeast form adherence to HSV-1- and HSV-2-infected cells was specific for the *Candida* phenotype tested. Our study suggests that HSV, while antagonist towards *S. aureus* adherence enhances *Candida* adherence. Furthermore, the combination of the three pathogens results in *S. aureus* adherence that is either unaffected, or partially restored depending on both the herpes viral species and the fungal phenotype present.

## Introduction

Adherence to cell surfaces is an essential initial stage in microbial colonization and subsequent biofilm formation [58, 77]. Shared sites of persistent colonization and chronic infection for *Staphylococcus aureus,Candida albicans,* and HSV are the oronasopharynx and genital tract*. S. aureus* and *C. albicans,* both commensals, are also the 2nd and 4th most common cause of bloodstream infections, respectively [54, 79]. Of the various sites of *S. aureus* and *C. albicans* co-colonization, the oronasopharynx serves as the reservoir for systemic infections [44]. Within the oronasopharynx, *S. aureus,C. albicans*, and HSV occupy two distinct geographic niches. In hosts with dentition, the oral mucosa is shared by HSV and *C. albicans,* while the anterior nasal nares are occupied by *S. aureus* [21]. Clinically, *S. aureus* is only rarely isolated from oral-pharyngeal specimens when normal tissue is present, despite in vitro findings that *S. aureus* adheres to buccal epithelial cells [22, 51]. Interestingly, in the presence of dentures, an abiotic surface, *S. aureus* forms a robust biofilm on the denture surface along with *C. albicans* [29, 63]. Little is known concerning genital tract co-colonization niches beyond the clinical findings that *S. aureus* infection is associated with genital inflammation, discharge, and dyspareunia, while *C. albicans* and HSV produce mucosal lesions similar to those observed in the oral cavity [25, 39, 46, 55, 59]. Whether present in the oronasopharynx or genital tract, it is a near certainty that *S. aureus* and *C. albicans* would interact at some point with HSV, a permanent resident of infection sites [5].

HSV, a major cause of morbidity and mortality, is a life-long pathogen present in >90 % of the world population [11, 71]. In immune-competent individuals, HSV-1 is a major cause of gingivostomatitis, as well as genital herpes, due to changes in sexual behaviors [5, 48]. Similar to HSV-1, HSV-2 causes oral lesions, although it has a higher association with genital lesions [7, 72]. A characteristic of herpes infections is chronic persistent viral shedding in the absence of symptoms [65, 67]. This permanent, albeit intermittent, presence of HSV virions may play a role in regulating the host microbiome. This could be accomplished via an alteration in available cell surface receptors for adherence by other members of the microbiome. [6, 8–10, 12, 16, 19, 21, 53]. Using a HeLa cell model of virus infection, the focus of this study was to determine whether HSV-1 or HSV-2 affect *S. aureus* and/or *C. albicans* germ tube and yeast form adherence, the initial step in biofilm formation.

## Methods

### Microbial Strains and Handling

Recombinant spread-deficient, entry proficient strains of HSV-1(KOS) gL86 and HSV-2 (KOS) 333gJ^−^ encoding a beta-galactosidase reporter activity were used [3, 37, 68, 74]. Both virus strains enter and replicate, thus have the potential to induce cell signaling, but lack the genes essential for viral cell-to-cell spread. All virus used in this study were taken from a single lot. Virus stocks were maintained at −80 °C until use. HSV entry into HeLa cells was confirmed by *o*-nitrophenyl-*β*-d-galactopyranoside (ONPG) and 5-bromo-4-chloro-3-indolyl-*β*-d-galactopyranoside (X-Gal) assays, as previously described [52]. HeLa 229 cells were maintained and cultivated at 37  °C, 5 % CO_2_ in 1× Dulbecco’s modified Eagle’s medium (DMEM with 4.5 g/L glucose and l-glutamine, without sodium pyruvate; Mediatech) supplemented with 10 % heat-inactivated fetal bovine serum (FBS) and gentamicin (50 µg/ml).

*Candida albicans*, maintained at −80 °C until use, was initially subcultured onto Sabourad Dextrose agar. For use, *C. albicans* was cultured onto Fungisel medium (37 °C; 48 h;Troy Biologics). Yeast suspensions (YF) were prepared in Hanks Balanced Salts Solution (HBSS; 10^5^ CFU/ml final concentration; 37 °C) immediately prior to use. Germ tube forms (GT) were generated by incubation in fetal bovine serum (FBS; 3 h; 37 °C; Abs_600_ 0.3), followed by washing in HBSS (2×; 4000×*g*) and re-suspension in HBSS to 10^5^ CFU/ml final concentration.

*Staphylococcus aureus* ATCC 25923, maintained at −80 °C until use, was subcultured onto mannitol salts medium (37 °C; 18 h) for use. *S. aureus* suspensions were prepared immediately prior to use in HBSS (10^5^ CFU/ml final concentration; 37 °C).

### Polymicrobic Adherence Assay

The number of HeLa cell-associated *S. aureus* and *C. albicans* was determined as an indicator of biofilm initiation (adherence). HeLa 229 were grown overnight in 96-well (4 × 10^4^ cells/well) at 37  °C, 5 % CO_2_ to reach 85 % final confluence. Before infection with virus, the cells were washed with 1× Opti-MEM with HEPES, sodium bicarbonate and l-glutamine (Gibco). Virus (HSV-1 (KOS) gL86 or HSV-2 (KOS) 333gJ^**−**^) was added to HeLa 299 cells at a multiplicity of infection (MOI) of 50 and 10 for 3 h at 37  °C, 5 % CO_2_. After viral infection, the cells were washed once each with PBS then HBSS before incubation with *C. albicans* YF or GT with and without *S. aureus* (5:1 target to cell ratio; *n* = 16). After incubation (30 min; 37 °C; 5 % CO_2_), HeLa cell monolayers were washed to remove unbound microbes (PBSx3) and lysed (RIPA, Life Technologies, 1:50 dilution; filter sterilized). The cell lysate (50 µl) was spread plated onto mannitol salts and/or Fungisel agar to select for *S. aureus* and *C. albicans*, respectively. Controls consisted of HSV-uninfected HeLa cells handled as described for virus-infected HeLa cells. For each experiment, a separate control plate to confirm the viral MOI was performed.

Imaging studies of HSV-1- and HSV-2-infected (MOI 50; 3 h; 37 °C) HeLa cell monolayers (5 × 10^4^ cells/coverslip) were performed by both fluorescent microscopy and bright field microscopy. Cell monolayers were incubated (30 min; 37 °C; 5 % CO_2_) with *S. aureus* and *C. albicans* (5:1 target to cell). HeLa cells were then washed free of non-adherent microbes (PBSx3) and fixed (methanol). For fluorescent microscopy, monolayers were stained with FITC-conjugated Herpes Simplex Virus Type 1+2 gD antibody (Abcam), and 4′,6-diamidino-2-phenylindole (DAPI; Life Technologies) then examined by epi-fluorescent microscopy. Cells that were signal positive for HSV or *Candida* or *S. aureus* (100 individual cells per microbe signal per coverslip) were secondarily scanned for the presence of additional microbe co-localization signals (1000× initial magnification; Nikon). For bright field microscopy, cell monolayers were stained with Gram’s crystal violet (Troy Biologics), then examined by light microscopy. Cells that were positive for *Candida* or *S. aureus* (100 individual cells per microbe signal per coverslip) were secondarily scanned for the presence of additional microbe co-localization signals (1000× initial magnification).

### Statistical Analysis

Each adherence experiment was conducted twice in octuplicate. Each imaging study was conducted twice in triplicate. Data were evaluated by analysis of variance (ANOVA; GraphPad InStat 3.10 for Windows, GraphPad Software Inc.). Mean values were considered significantly different at *P* < 0.05.

## Results

### *Candida,* HSV-1 and HSV-2 Modulation of *S. aureus* Adherence to HeLa Cells

**Fig. 1:**
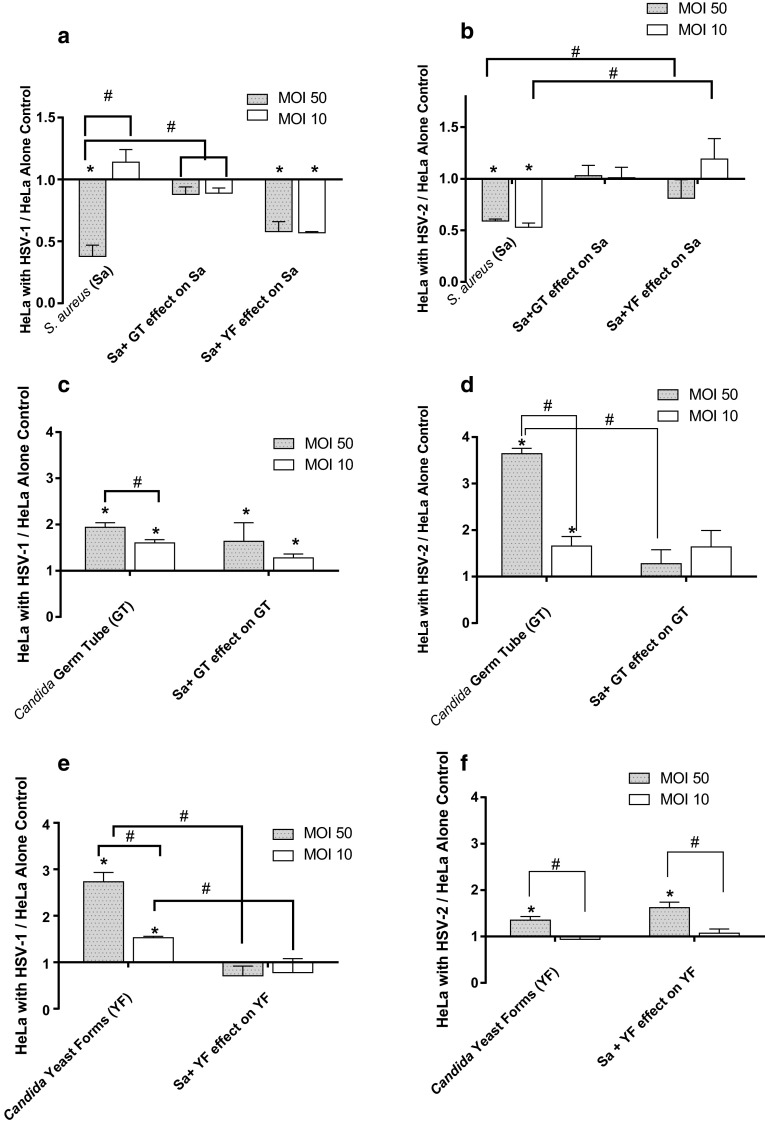
Effect of HSV-1 (panels **a**, **c**, **e**) and HSV-2 (panels **b**, **d**, **f**) at multiplicities of infection (MOI) of 50 and 10 on adherence of *S. aureus* and/or *C. albicans* to HeLa cells. **a** *S. aureus* (Sa) binding to HSV-1-infected cells in the presence of *C. albicans* germ tubes (GT) or yeast forms (YF); **b** *S. aureus* (Sa) binding to HSV-2-infected cells in the presence of *C. albicans* germ tubes (GT) or yeast forms (YF); **c** *C. albicans* germ tubes (GT) binding to HSV-1-infected cells in the presence of *S. aureus* (Sa); **d** *C. albicans* germ tubes (GT) binding to HSV-2-infected cells in the presence of *S. aureus* (Sa); **e** *C. albicans* yeast forms (YF) binding to HSV-1-infected cells in the presence of *S. aureus* (Sa); **f** *C. albicans* yeast forms (YF) binding to HSV-2-infected cells in the presence of *S. aureus* (Sa). All data points are mean ± SEM, *n* = 16 normalized to virus-free control. *Significantly different (*P* < 0.05) from uninfected HeLa cell control. ^#^Significantly different (*P* < 0.05) from paired point indicated by bracket

Since HSV-1 and HSV-2 co-localize in the oropharynx with *C. albicans*, we first determined their effect alone, and in concert, on *S. aureus* adherence to virus-infected HeLa cells as compared to *S. aureus* adherence to virus-free HeLa cells. Both HSV-1- and HSV-2-affected *S. aureus* adherence to HeLa cells (Fig. 1a, b). At the highest virus concentration (MOI 50) the level of *S. aureus* adhered to uninfected HeLa cells (24.2 CFU/well ± 3.19; *n* = 16) was significantly higher (2.7-fold; *P* < 0.05) than that measured for *S. aureus* adherence to HSV-1-infected HeLa cells (9.1 ± 1.1 CFU/well). Staphylococcal adherence to uninfected HeLa cells was also significantly (1.7-fold; *P* < 0.05) higher than that measured for adherence to HSV-2 (MOI 50)-infected HeLa cells. The level of *S. aureus* adherence to HSV-2-infected cells at a fivefold lower MOI (MOI 10) was similar to that measured for the HSV-2 MOI 50 (1.9-fold below virus-free control).

Co-incubation of *S. aureus* with *C. albicans* germ tube (GT) forms restored the level of *S. aureus* adherence to HSV-1 and HSV-2-infected HeLa cells to that measured for uninfected HeLa cell controls. Similar to the effect of GT forms, *S. aureus* adherence to HSV-2-infected cells in the presence of *C. albicans* yeast forms (YF) resulted in staphylococcal adherence levels similar to that measured uninfected HeLa cells. In contrast, the presence of YF did not affect the HSV-1 mediated depression in *S. aureus* adherence. These results show that HSV viral entry is antagonist to *S. aureus* adherence, an antagonism that can be, in part, reversed by *C. albicans* GT and YF.

### *C. albicans* Adherence to HSV-1- and HSV-2-Infected HeLa Cells in the Presence and Absence of *S. aureus*

In these assays, we determined whether *C. albicans* GT and YF adherence to HeLa cells was affected by HSV and *S. aureus* (Fig. 1c–f). Our findings showed that HSV-1 and HSV-2 significantly (*P* < 0.05) enhanced *C. albicans* GT and YF adherence in a virus dose-dependent manner; maximal adherence levels of GT and YF occurred at an MOI of 50. For both candidal phenotypes, adherence was significantly higher (*P* < 0.05) than that measured for adherence to virus-uninfected HeLa cell controls. *Candida* GT adherence to HSV-2-infected HeLa cells (normalized to virus-uninfected HeLa cell controls) was significantly (*P* < 0.05) higher than its adherence to HSV-1-infected HeLa cells (3.6-fold vs. 1.9-fold higher, respectively). The reverse pattern was observed for YF adherence. YF significantly (*P* < 0.05) and preferentially adhered to HSV-1-infected cells (2.7-fold over uninfected control) as compared to YF adherence to HSV-2-infected cells (1.9-fold over uninfected control).

The HSV-mediated enhancement of YF and GT adherence was significantly (*P* < 0.05) and preferentially affected by the presence of *S. aureus*. *S. aureus* decreased GT adherence to HSV-2-infected cells to levels measured for uninfected HeLa cell control. In contrast, *S. aureus* significantly (*P* < 0.05) decreased YF adherence to HSV-1-infected HeLa cells. *S. aureus* had no significant effect on YF adherence to HSV-2-infected cells or GT adherence to HSV-1-infected HeLa cells. Taken together, these results indicate that HSV promotes the association of *C. albicans* in a phenotypic-specific manner. Furthermore, these findings indicate that *S. aureus’* effect on *C. albicans* adherence was HSV-type specific with regard to fungal phenotype.

### Microscopic Evaluation of *C. albicans* and *S. aureus* Adherence Pattern to HSV-1- and HSV-2-Infected HeLa Cells

**Fig. 2:**
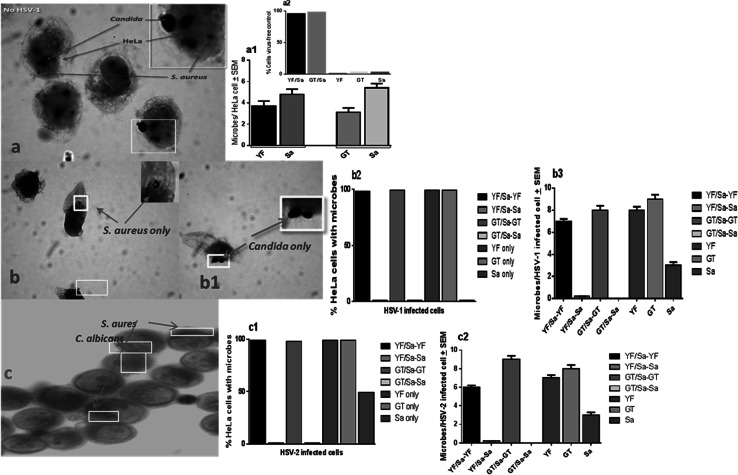
Lack of *S. aureus* and *C. albicans* interactions on HSV-1- and HSV-2-infected HeLa cells. HSV-1 and HSV-2-infected (MOI 50) HeLa cell monolayers with *S. aureus* and *C. albicans* (5:1 target to cell). For bright field microscopy, cell monolayers were stained with Gram’s crystal violet, then examined by light microscopy. Cells that were positive for *Candida* or *S. aureus* (100 individual cells per microbe signal/per coverslip) for were secondarily scanned for the presence of additional microbe co-localization signals (×1000 initial magnification). * a*—*a1* *S. aureus* (Sa) and *C. albicans* yeast forms (YF) or germ tube forms (GT) co-localize on uninfected HeLa cells; *a2* (insert) Percent of HeLa cells with co-localized or individual microbes; * b*—*b3* Lack of *S. aureus* and *C. albicans* co-localization in the presence of HSV-1; * c*—*c2* Lack of *S. aureus* and *C. albicans* co-localization in the presence of HSV-2; mean ± SEM

**Fig. 3:**
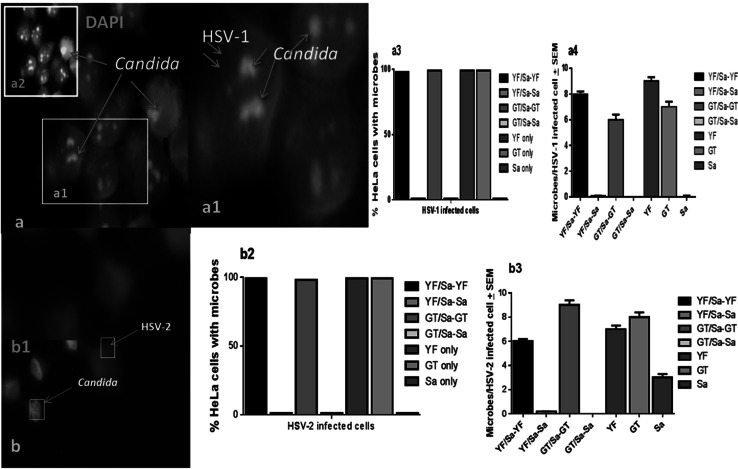
Lack of co-localization of *S. aureus* with *C. albicans* on HSV-1- or HSV-2-infected HeLa cells. HSV-infected HeLa cell monolayers challenge with *S. aureus* and *C. albicans* (5:1 target to cell) stained (FITC-conjugated anti-HSV gD antibody, and DAPI). Pictures are representative of findings from screening of cells that were signal positive for HSV then scanned for *Candida* and *S. aureus* (100 individual cells per microbe signal per coverslip) that were then secondarily scanned for the presence of additional microbe co-localization signals (×1000 initial magnification; Nikon). * a*—*a4* *C. albicans* (*a2* insert; DAPI staining) co-localize with HSV-1; * b*-*b3* *C. albicans* co-localized with HSV-2 (*b1* insert, *C*. *albicans* DAPI staining); mean ± SEM

We visually examined the association of *S. aureus* and *C. albicans* with HSV-1- and HSV-2-infected HeLa cells using the size, morphology, and arrangement differences between *S. aureus* and *C. albicans* to distinguish between the organisms. In the absence of HSV-1 or HSV-2, *S. aureus* and *C. albicans* (GT and YF) co-localized onto uninfected HeLa control cells (Fig. 2a). In contrast, no co-localization of staphylococci with *C. albicans* was observed on HSV-1- or HSV-2-infected HeLa cells (Fig. 2b–d). Using fluorescent microscopy with FITC-conjugated anti-HSV-gD monoclonal antibody, we further confirmed that *S. aureus* (Fig. 3b, c) did not co-localize with *C. albicans,* HSV-1 or HSV-2 (Fig. 3a, a1, 3d). However, *Candida* co-localized with HSV-1 and HSV-2 (Fig. 3a1, c). *S. aureus* and *C. albicans* were not observed microscopically to interact with one another (data not shown). Furthermore, the co-localization adherence pattern of *S. aureus* and *Candida,* in the absence of HSV, indicates a lack of physical interference with each other’s adherence. These findings further suggest a depletion of available *S. aureus* adherence receptors subsequent to HSV cell entry.

## Discussion

In this report, we describe an in vitro model system to study the polymicrobial interactions which occur at the initiating step in biofilm formation, i.e., adherence. The use of HeLa cells in this model system presents a unique advantage in the study of microbial interactions regarding cell surface receptors as they lack surface expression of fibronectin [4, 13, 27]. This absence of surface fibronectin more closely mimics that observed in colonization and natural infection, since the apical surface of mucosal epithelia normally lacks fibronectin [18, 33, 38, 49, 66]. This absence of fibronectin also allows for the study of alternative mechanisms by which *S. aureus* and *C. albicans* adhere to HSV-infected cells since both microbes adhere to fibronectin [2, 4, 13, 40, 69]. This model system for the study of virus effects on bacterial and fungal adherence was further enhanced through the use of entry and replication proficient, but non-spreading strains of HSV-1 and HSV-2 [3, 74].

**Fig. 4:**
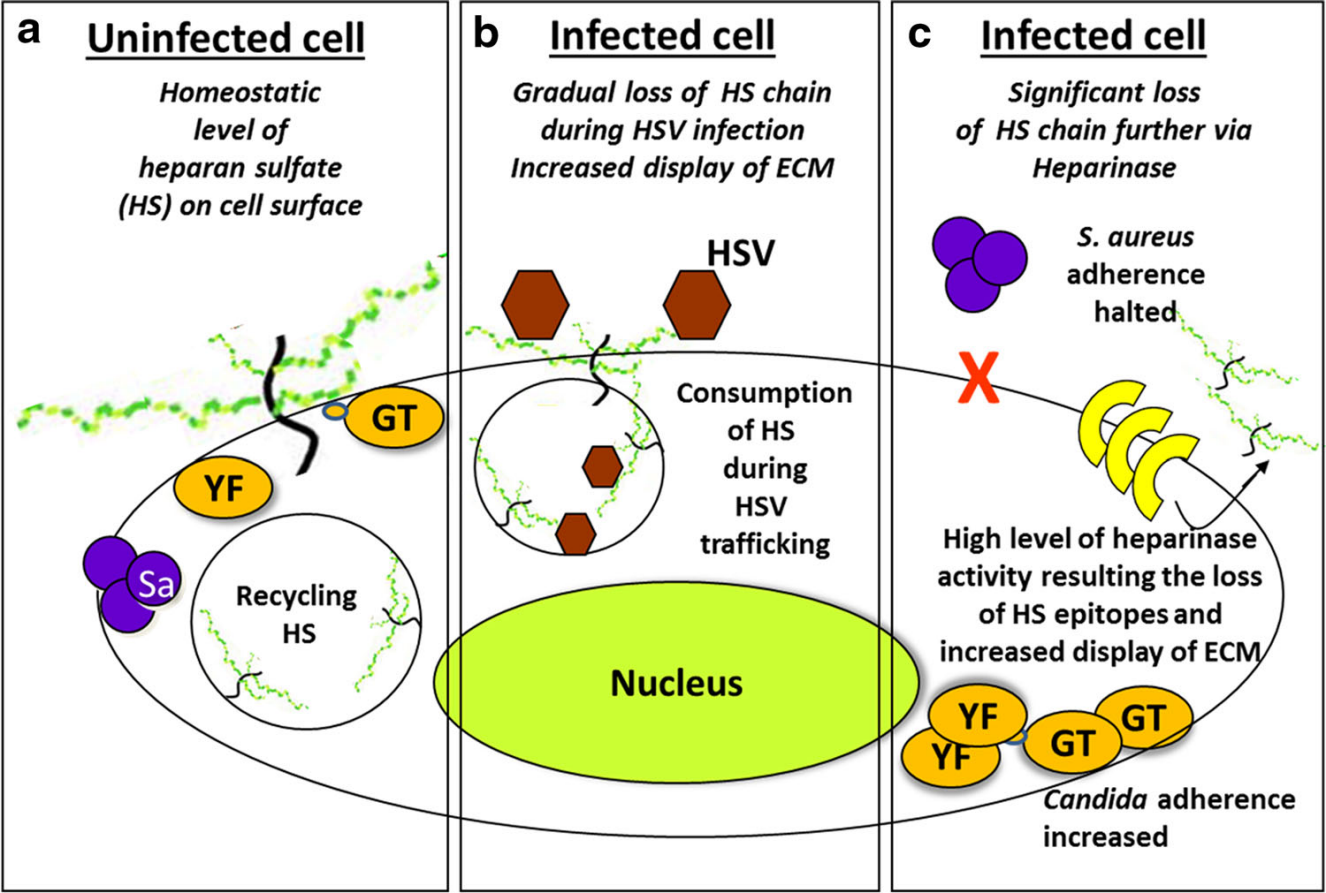
Proposed model of the key steps involved in polymicrobic interaction (see text for details). *Sa* *S. aureus*, *YF* yeast form, *GT* germ tube form, *HS* heparan sulfate, *HSV* herpes simplex virus, *ECM* extracellular matrix proteins other than glycosaminoglycans, e.g., laminin and collagen

HSV enters cells via endocytosis [56, 57]. Endocytosis of the virus-receptor complex results in a dynamic display of cell surface receptors available to *S. aureus* and *C. albicans* for their adherence. Interestingly there is an opposing interaction of *S. aureus* and *C. albicans* with heparan sulfates on the mammalian cell surface. *S. aureus* adherence to various sulfonated heparans, particularly a heparan component syndecan-1, is important in its interaction with epithelial cells [31, 36, 42, 47, 61, 73]. In contrast, the presence of cell surface heparan sulfates block *C. albicans* adherence to extracellular matrix proteins, e.g., laminin, and types I and IV collagen [43]. HSV cell entry causes a species specific (HSV-1 vs. HSV-2) differential depletion of heparan sulfates, and induction of heparanase secretion, which further depletes cell surface sulfonated heparans [1, 14, 20, 28, 30, 35, 41, 45, 56, 57, 70, 75]. Both these events seem critical in our observations. For instance, loss of *S. aureus* heparan sulfate receptors could explain the lack of its adherence to HeLa cells infected with either HSV-1 or HSV-2 (Fig. 4). Conversely, *Candida* preferred receptors would be unmasked by heparanase production and HSV entry depletion of surface heparan sulfates. The HSV-1 vs. HSV-2 differential depletion of sulfonated heparan receptors could explain our findings of enhanced adherence to virus-infected cells that was *Candida* phenotype (YF-GT) dependent (Fig. 4) [45].

Although this model begins to address the patterns measured for *S. aureus* and *C. albicans* adherence to HSV-infected cells, a secondary mechanism is likely responsible for the effects of *S. aureus* and *C. albicans* on each other’s adherence in the presence of both uninfected and HSV-infected (50 % HeLa cells infected/well, MOI50) cells. HSV-infected cells release interferons alpha and gamma, which in turn cause perturbation of lipid rafts altering membrane fluidity (hydrophobicity) and the cell cytoskeletal complex in non-virally infected cells [24, 34, 60, 64, 72]. Both *S. aureus* and *C. albicans* interact with hydrophobic surfaces [2, 21, 32]. The secondary extrinsic HSV-mediated effects on uninfected cells, i.e., surface hydrophobicity changes, could result in increased *S. aureus* and *C. albicans* adherence and the apparent restoration of cell binding. The lack of cell-to-cell spread of the virus in this model system will allow for determination of which virally mediated cell alteration, i.e., intrinsic, extrinsic, or a combination of changes, is responsible for the differential adherence by *S. aureus* and *C. albicans* [2, 21].

The finding herein that HSV-1 and HSV-2 renders cells refractory to *S. aureus* adherence, while enhancing phenotype specific *C. albicans* adherence, as well as predilection for differential fungal phenotype adherence mediated by HSV-1 (yeast form) vs. HSV-2 (germ tube form) begin to explain host colonization sites and may play a role directing maintenance of the candidal commensal (YF) versus pathogenic state (GT) in vivo, e.g., HSV-2 may impact the incidence of vaginal candidiasis. These positive interactions between HSV and *C. albicans* may also provide an environment more conducive to HSV survival. Recently it has been shown that HSV-1 becomes embedded in the fungal biofilm where it remains viable and protected from antiviral agents, as well host factors, e.g., antibodies [50]. This protection of HSV from host factors is augmented by *Candida*’s ability to downregulate host proinflammatory cytokine secretion, which would be detrimental to HSV replication [17, 23, 26, 76]. HSV-1 in turn has been shown to protect *Candida* by down-regulating antifungal immune response [15]. Both HSV and *Candida* would benefit from the exclusion of *S. aureus* from their neighborhood. *S. aureus* is a proinflammatory pathogen [80]. In addition to the induction of various cytokines, including interferon gamma, the chronic presence of *S. aureus* elicits a leukocytic infiltrate comprised neutrophils, T cells and natural killer cells [62, 78]. Such a proinflammatory response would be detrimental to both HSV and candidal survival.

To our knowledge this is the first report demonstrating the ability of HSV to regulate the adherence of multiple opportunistic pathogens that are part of the inter-kingdom microbiome. This study demonstrates that HSV-1 and HSV-2 modulate both fungal and bacterial adherence to cells, likely, in part, through HSV alteration of heparan sulfate cell surface display. These findings represent a paradigm shift from the current view that host factors solely control microbiome population members, to one where a life-long latent viral pathogen could co-opt the host by a specific molecular mechanism that alters biofilm initiation, thus, usurping regulatory control of microbiome membership. Further studies are needed to define the specific role the various HSV viral entry receptors play in modulation of staphylococcal and candidal adherence. By understanding the initial interactions that occur between HSV-1 and HSV-2 as permanent members of the host microbiome, and chronic colonizers, e.g., *S. aureus* and *C. albicans,* another avenue opens with regard to biofilm inhibition and eradication.
